# Prevalence of hepatitis B and hepatitis C among diabetes mellitus type 2 individuals

**DOI:** 10.1371/journal.pone.0211193

**Published:** 2019-02-28

**Authors:** Livia Melo Villar, Bruno Geloneze, Ana Carolina Junqueira Vasques, Maria Lucia Elias Pires, Juliana Custódio Miguel, Elisangela Ferreira da Silva, Vanessa Alves Marques, Leticia de Paula Scalioni, Elisabeth Lampe

**Affiliations:** 1 Laboratory of Viral Hepatitis, Oswaldo Cruz Institute, FIOCRUZ, Rio de Janeiro, Rio de Janeiro State, Brazil; 2 Laboratory of Investigation in Metabolism and Diabetes (LIMED), Gastrocentro, University of Campinas (UNICAMP), Campinas, Sao Paulo, Brazil; 3 School of Applied Sciences, University of Campinas, Limeira, Sao Paulo, Brazil; 4 Department of Clinical Medicine of Surgery and Medicine School, Federal University of State of Rio de Janeiro/UNIRIO, Rio de Janeiro, Rio de Janeiro State, Brazil; The University of Tokyo, JAPAN

## Abstract

Diabetes mellitus type 2 (DM2) patients have higher risk to be infected with parenterally transmitted viruses, like hepatitis B or C virus. This study aims to determine HBV and HCV infection prevalence in DM2 patients from Northeast and Southeast Brazil. A total of 537 DM2 patients were included, 194 (36.12%) males and 343 (63.87%) females, with mean age of 57.13±11.49 years. HBV and HCV markers were determined using serological and molecular analysis, and risk factors were evaluated in a subgroup from Southeast (n = 84). Two HBV acute (HBsAg+/anti-HBc -) and one HBV chronic case (HBsAg+/anti-HBc+) were found. Six individuals (1.1%) were isolated anti-HBc, 37 (6.9%) had HBV infection resolved (anti-HBc+/anti-HBs+), 40 (7.4%) were considered HBV vaccinated (anti-HBc-/anti-HBs+). Thirteen patients (2.42%) had anti-HCV and 7 of them were HCV RNA+. In the subgroup, anti-HBc positivity was associated to age and anti-HCV positivity was associated to age, time of diabetes diagnosis, total bilirubin, indirect bilirubin, alkaline phosphatase at bivariate analysis, but none of them was statistically significant at multivariate analysis. As conclusion, low prevalence of HBV and high prevalence HCV was found in DM2 patients.

## Introduction

Hepatitis B and C virus infection are major global health problems. All over the world, it has been estimated that 257 million persons are chronically infected with HBV and 71 million of individuals are HCV chronic carriers [1,2]. In Brazil, overall prevalence varies from 0 to 16.8% for Hepatitis B surface antigen (HBsAg) and 1.38% up to 47% for antibodies against HCV (anti-HCV) according geographic region or specific groups[3–8].

Diabetes mellitus type II (DM2) is a major public health problem in Brazil and is one of the fastest growing diseases around the world. The International Diabetes Federation estimates there are now 425 million adults aged 20–79 with diabetes worldwide, including 212.4 million who are undiagnosed. In 2045, the estimate is that there are about of 629 million of people with DM. Currently, Brazil occupies the first place among the countries of South America, with 13 million individuals with diabetes and occupies the fourth position among the 10 countries with the highest number of adults with DM2 / territory [9].

According to Brazilian National Household Sample Survey (PNAD), the prevalence of diabetes in older individuals has been increased from 10% to 16% in the period of 1998 to 2008 in Brazil [10].

Liver is the principal site of hormone and glucose metabolism and about 30% of patients with cirrhosis have diabetes mellitus type 2 [11]. DM2 patients have higher risk to be infected with parenterally transmitted viruses, like hepatitis B or C virus since they undergo frequent hospitalization and are submitted to blood tests, like blood glucose monitoring [12].

Hepatitis B infection outbreaks were reported in people/patients with diabetes due to misuse of fingerstick devices for monitoring the capillary blood glucose level [13]. HCV infection also could be transmitted due to frequent exposure to invasive procedures [14].

HCV prevalence in DM2 patients vary from 1.6 to 20.8% according geographical regions and presence of comorbidities like hemodialysis [12,14,15–17]. Naing et al.[18] observed high prevalence of diabetes mellitus type 2 in hepatitis C-infected patients compared to uninfected controls.

A study in China demonstrated higher prevalence of HBV infection (13.5%) in diabetes mellitus type 2 compared to controls (10.0%) [19]. Indeed, a recent metanalysis of studies from North America and Asia demonstrated that 8.2% of HBV-infected patients also suffered from DM2 [20].

In Brazil, anti-HCV prevalence varied from 2 to 7% in DM patients from Central West and South region of Brazil [21–23] while DM2 was observed in 10.3% of renal transplant patients infected with HBV in Brazil [24]. At this moment, little is known about the prevalence of these viruses in other regions of Brazil, the risk factors related to HBV and HCV positivity, HBV and HCV viremia and genotypes in these individuals. The objectives of this study were to estimate the seroprevalence rates of hepatitis B and C in DM2 patients from Northeast and Southeast regions of Brazil, to explore the risk factors for hepatitis in a sub group of DM2 patients from Southeast region (Rio de Janeiro State) and to determine HBV and HCV viremia.

## Material and methods

### Population studied

This was a cross-sectional study on DM2 patients recruited during 2007–2013 at two geographical regions in Brazil (Southeast and Northeast Region). Patients were recruited at endocrinology outpatient units in Federal University of State of Rio de Janeiro (UNIRIO-Rio de Janeiro) (n = 84) (Group 1) and from Brazilian Metabolic Syndrome Study (BRAMS), a multicenter survey, which included individuals from three different States in Brazil: Ceará (Northeast region), São Paulo and Minas Gerais (Southeast region) (n = 453) (Group 2). The subjects who were invited to participate were selected from outpatient clinics for the metabolic syndrome and obesity or through local and internet advertisements.

In this study, samples were selected using an intentional non-probabilistic sampling. A total of 537 DM2 patients were enrolled, 194 (36.12%) males and 343 (63.87%) females, with a median age of 57 years, ranged 29–89 years old. Data such as gender and age were collected from the clinical records of each patient from individuals from Ceará, Minas Gerais and São Paulo State. From Rio de Janeiro State, it was administered a questionnaire to collect information concerning gender, age, education level and risk factors for viral hepatitis such as sexual behavior and practices, occupational exposure, parenteral exposure to blood on previous transfusion, surgery, intravenous drug use and needle sharing.

The criteria for enrollment for both groups were diagnosis of DM if there was documented use of oral hypoglycemic medication or insulin or according to the American Diabetes Association criteria with random glucose more than 200 mg/dL, or fasting glucose greater than 126 mg/dL on two occasions. The exclusion criteria for both groups were the evidence of hepatocellular carcinoma and absence to consent.

The study protocol was carried out in accordance with the Declaration of Helsinki and was approved by the Ethics Committee of Oswaldo Cruz Foundation, Campinas University and Federal University of Rio de Janeiro State under the numbers of CAAE: 24930813.0.0000.5248; 24930813.0.3002.5404; 24930813.0.3001.5258.

All participants were given a verbal explanation of the objectives and methodology of the research and were included in the study after obtaining signed informed consent. All individuals who tested positive were sent to public health units to had access to treatment.

### Laboratory tests

Blood sample was obtained by venipuncture before the attendance at outpatient clinics. Serum samples from all individuals of the study were tested for HBsAg, anti-HBc, anti-HBs, anti-HCV using commercial eletrochimioluminescence assay (ECLIA) (Roche, USA) according to the manufacturer’s guidelines. Samples found to be negative on the preliminary screening were considered seronegative. Samples that initially tested borderline or positive were retested using ECLIA to confirm the results.

Serum aminotransferases (alanine aminotransferase [ALT] and aspartate aminotransferase [AST]), gamma-glutamyltransferase (GGT), phosphatase alkalin, total cholesterol, high-density lipoprotein (HDL) and low-density lipoprotein cholesterol (LDL), triglycerides and plasma glucose were measured with an automatic analyzer (Labmax 240 Premium, Labtest, Minas Gerais, Brazil). ALT, AST, GGT and glucose were determined in all individuals while these biochemical data, complete blood count, phosphatase alkalin, cholesterol, HDL cholesterol, Triglycerides, Bilirrubin total, direct and indirect were determined only from individuals from Rio de Janeiro State.

Serum HCV RNA and HBV DNA was detected in anti-HCV and HBsAg positive samples using a standardized automated quantitative assay (COBAS TaqMan HCV Test and HBV Test, Roche, Branchburg, NJ, USA) and expressed as IU/mL. The limit of detection is 3.5 IU/mL and 18IU/mL for serum by PROBIT analysis for COBAS TaqMan HBV Test and HCV Test, respectively.

### Data collection and analysis

Data are expressed as frequencies and mean with standard deviation. Bivariate analysis was performed using the chi-square (x2) test for independence with Yate’s continuity correction, x2 for trend and Fisher’s exact test when appropriate to compare proportions. Student’s t-test was used to analyse normally distributed quantitative variables and non-parametric (Mann-Whitney U test) statistics was used to analyse quantitative variables, which did not pass normality test (Kolmogorov-Smirnov test). Variables selected for their statistical significance (P < 0.05) in the bivariate analysis were entered the multiple logistic regression model in a stepwise fashion. Estimated odds ratio (OR) were also calculated. All calculations, including multiple logistic regression analysis (MLR), were undertaken by using the Statistical Package for the Social Sciences (SPSS for Windows, release 20.0; SPSS, Inc., Chicago, IL, USA).

## Results

### HBV and HCV markers in population studied

Among the 537 DM2 patients, 3 patients (0.55%) had HBsAg but no one had HBV DNA, 44 (8.19%) had previous contact to HBV (anti-HBc reactive), 77 (14.33%) were immune to HBV (anti-HBs reactive). In the group of HBV immune individuals, 40 (7.44%) were vaccinated (only anti-HBs reactive). Thirteen individuals (2.42%) had anti-HCV and seven of them were HCV RNA positive. One individual was HBsAg and anti-HCV reactive. A total of 452 individuals (84.17%) were seronegative for all HBV markers, but 8 of them were anti-HCV reactive. Age, gender and laboratory data from total population and groups 1 and 2 are presented in Table 1.

**Table 1:** Demographic, laboratory, HBV and HCV markers in type 2 diabetes mellitus patients recruited in groups 1 and 2.

Variable	Total Population (n = 537)	Group 2 (n = 453)	Group 1 (n = 84)
**Age, years** (mean±SD)	57.13±11.49	55.68±11.12	64.9±10.24
**Gender,** Male/Female (n)	194/343	172/281	22/62
**Platelet Count x 10**^**3**^**/mm,** (mean±SD)	206.60±47.99	ND	206.60±47.99
**ALT, IU/L,** (mean±SD)	25.18±26.26	26.76±27.37	14.56±12.72
**AST, IU/L,** (mean±SD)	22.57±13.20	23.24±12.93	17.92±14.23
**Phosphatase alkaline, IU/L,** (mean±SD)	64.90±27.33	ND	64.90±27.33
**GGT, IU/L,** (mean±SD)	42.87±40.00	44.53±41.39	31.14±25.63
**Cholesterol, mg/dL,** (mean±SD)	187.77±49.15	ND	187.77±49.15
**HDL cholesterol, mg/dL,** (mean±SD)	42.49±11.12	ND	42.49±11.12
**Triglycerides, mg/dL,** (mean±SD)	133.16±56.14	ND	133.16±56.14
**Total Bilirubin, ng/mL,** (mean±SD)	0.09±0.08	ND	0.09±0.08
**Indirect Bilirubin, ng/mL,** (mean±SD)	0.01±0.10	ND	0.01±0.10
**Direct Bilirubin, ng/mL,** (mean±SD)	0.11±0.09	ND	0.11±0.09
**Blood glucose, ng/mL,** (mean±SD)	153.72± 66.28	158.68±66.85	126.62±56.13
**Haemoglobin,** (mean±SD)	12.72±1.81	ND	12.72±1.81
**HBsAg reactive, n (%)**	3 (0.55)	3 (0.66)	0 (0.00)
**Anti-HBc reactive, n (%)**	44 (8.19)	28 (6.18)	16 (19.04)
**Anti-HBs reactive, n (%)**	77 (14.33)	52 (11.47)	25 (29.76)
**Anti-HBs titer, IU/mL,** (mean±SD)	183.30±272.36	69.07±35.72	407.51±379.53
**Anti-HCV, n (%)**	13 (2.42)	7 (1.54)	6 (7.14)

ND: not done

In group 1, high mean±SD age (64.90±10.24 years) and females (73.8%) were predominant. Previous HBV infection and HBV immunity were more frequent in this group as determined by anti-HBc (19.04%) and anti-HBs (29.76%) positivity, respectively. Anti-HCV prevalence was high in group 1 (7.14%), although only 2 were HCV RNA reactive.

Among group 2 (individuals from Minas Gerais, Ceará and São Paulo State), 172 (37.96%) males and 281 (62.03%) females and mean±SD age of 55.68±11.12 years. In this group, 3 had HBsAg, 28 were anti-HBc reactive, 52 were anti-HBs reactive, 7 were anti-HCV reactive and 5 out 7 were HCV RNA reactive. High mean values of AST, ALT, GGT and blood glucose was observed in this group compared to group 1.

### Demographic and risk factor characteristics in group 1

The socio-demographic characteristics and risk factors of the 84 individuals with diabetes from Rio de Janeiro are shown in Table 2. Most of participants in this study were females (73.8%), married (52.38%), had preschool (36.90%), received from 294.78–884.38 US dollars, had mean age of 64.99±10.24 years and more than 10 years of diabetes diagnosis (48.80%).

**Table 2:** Demographic and risk factors related to anti-HBV antibodies (total anti-HBc and anti-HBs) and anti-HCV positivity among person with diabetes in group 1 (*n* = 84).

Items	Total n = 84 (%)	Anti-HBc positive n = 16 (%)	Anti-HBs positive n = 25 (%)	Anti-HCV positive n = 06 (%)
Age, mean±SD	64.99±10.24	67	64	56
Gender, Female/Male	62/22	10/6	19/6	5/1
Marital Status, married	44 (52.38)	10 (62.5)	13 (52.00)	2 (33.33)
Scholarity Illiterate	4 (4.76)	0 (0.0)	0 (0.00)	0 (0.00)
Pre-school	31 (36.90)	13 (81.25)	12 (48.00)	1 (16.66)
Primary School	28 (33.33)	3 (18.75)	8 (32.00)	3 (50.00)
Secondary School	15 (17.85)	0 (0.0)	1 (4.00)	1 (16.66)
College	6 (7.14)	0 (0.0)	3 (12.00)	1 (16.66)
Family Income Nothing	2 (2.38)	1 (6.25)	1 (4.00)	0 (0.00)
U$S 294.78–884.38	35 (41.66)	8 (50.0)	12 (48.00)	2 (33.33)
More than U$S 884.38	9 (9.52)	1 (6.25)	3 (12.00)	1 (16.66)
Dark urine and hepatitis confirmed	13 (15.47)	4 (25.00)	6 (24.00)	3 (50.00)
HBV Vaccine	06 (7.14)	0 (0.00)	3 (12.00)	1 (16.66)
Blood transfusion	14 (16.66)	5 (31.25)	7 (28.00)	3 (50.00)
Haemodialysis	02 (2.38)	0 (0.00)	1 (4.00)	1 (16.66)
Acupuncture	18 (21.42)	2 (12.5)	6 (24.00)	1 (16.66)
Sharing razors/blade	07 (8.33)	1 (6.25)	3 (12.00)	0 (0.00)
Sharing Toothbrushes	15 (17.85)	3 (18.75)	6 (24.00)	3 (50.00)
Venous access	69 (82.14)	14 (87.5)	20 (80.00)	5 (83.33)
Manicure	58 (69.04)	10 (62.5)	18 (72.00)	4 (66.66)
Number of sexual partners Regular	43 (51.19)	5 (31.25)	13 (52.00)	5 (83.33)
Less than 5	28 (33.33)	7 (43.75)	7 (28.00)	1 (16.66)
No sexual partner	13 (15.47)	4 (25.00)	5 (20.00)	0 (0.00)
Condom usage				
Rarely	11 (13.09)	3 (18.75)	3 (12.00)	1 (16.66)
Never	50 (59.52)	7 (43.75)	12 (48.00)	3 (50.00)
Always	6 (7.14)	1 (6.25)	3 (12.00)	1 (16.66)
Oral sexual intercourse	33 (39.28)	6 (37.5)	7 (28.00)	3 (50.00)
Anal sexual intercourse	13 (15.47)	3 (18.75)	4 (16.00)	3 (50.00)
Contact with person with hepatitis	21 (25.00)	0 (0.00)	0 (0.00)	1 (16.66)
Infection Sexually transmitted (IST)	14 (16.66)	6 (37.5)	5 (31.25)	0 (0.00)
Drinking alcohol	16 (19.04)	3 (18.75)	7 (28.00)	1 (16.66)
Time of Diabetes diagnosis				
<1 year	03 (3.57)	0 (0.00)	1 (4.00)	2 (33.33)
1–5 years	21 (25.00)	3 (18.75)	7 (28.00)	0 (0.00)
5–10 years	15 (17.85)	4 (25.00)	6 (24.00)	0 (0.00)
>10 years	41 (48.80)	9 (56.25)	10 (40.00)	3 (50.00)

Thirteen individuals reported a previous history of hepatitis, and six reported HBV vaccination. Regarding risk factors, only two were under hemodialysis and one had tattoo. Fourteen individuals had received a blood transfusion, 8 of which were received before 1994. Most of these individuals visited manicurists (69.04%), but 16 of them (27.3%) used their own nail plier. Most of individuals had history of venous access (82.14%) and no one reported drug use. Regarding sexual orientation, 76 (90.47%) of these individuals were heterosexual, 1 (1.19%) was bisexual and 07 (8.33%) did not informed. Forty-three (51.19%) had a regular partner, 61 (63.0%) individuals never or rarely used condoms during sexual intercourse, 33 (39.28%) reported engaging in oral sex and 13 (15.47%) in anal sex, 14 (16.66%) had a history of IST, and four (4.76%) had a sexual partner infected with HIV or viral hepatitis.

### Demographic and laboratorial data according HCV infection in group 2

Anti-HCV positivity was evaluated regarding gender, age, and biochemical characteristics (Table 3). In this group, high mean values of GGT, AST, ALT were found among HCV reactive individuals. In addition, anti-HCV cases were from São Paulo and Ceará States (Southeast and Northeast region). Anti-HCV was associated to ALT values in bivariate analysis (p = 0.017).

**Table 3:** Bivariate analysis of factors associated to anti-HCV positivity in group 2.

Variable	anti-HCV		Bivariate analysis P Value
	Negative (n = 446)	Positive (n = 07)	
**Age,** mean±SD **years**	55.68 ±11.18	55.57 ±7.57	0.928
**Gender**			0.148
Female	279	02	
Male	167	05	
**Glucose (mg/dL),** (mean±SD)	158.89 ±67.05	146.14±56.11	0.612
**Insulin (μU/mL),** (mean±SD)	15.48 ±19.85	10.19 ±7.70	0.181
**GGT (IU/L),** (mean±SD)	44.05 ±41.04	75.57 ±54.98	0.057
**AST (IU/L),** (mean±SD)	23.15±12.86	29.14 ±17.31	0.167
**ALT (IU/L),** (mean±SD)	26.57 ±27.49	39.14 ±15.24	**0.017**
**PCR,** (mean±SD)	0.55 ±0.79	0.14 ±0.12	0.141
**State of Brazil**			0.064
Ceará	240	03	
Minas Gerais	120	00	
São Paulo	86	04	

NA: Not available.

Values in bold indicate significant values (p < 0.05).

### HBV and HCV prevalence and risk factors in group 1

Anti-HBc and anti-HBs prevalences were 19.04% and 29.76%, respectively. Thirteen individuals (15.47%) were HBV vaccinated, 12 (14.3%) individuals were resolved HBV infection and four were anti-HBc isolated (4.8%). Using anti-HBc as dependent variable in bivariate analysis, only age was statistically significant.

In group 1, anti-HCV prevalence was 7.14%. In bivariate analysis, anti-HCV positivity was associated with age, time of diabetes diagnosis, total bilirubin, indirect bilirubin, alkaline phosphatase, but none of them was found to be statistically significant in the multivariate analyses (Table 4).

**Table 4:** Bivariate and multivariate analysis of demographic and laboratorial data associated with anti-HCV prevalence in group 1.

Variable	Total Anti-HCV		Bivariate analysis *P* Value	Multivariate analysis OR	*P* value
	Reactive (n = 06)	Non reactive (n = 78)			
**Age, years,***mean±SD*	42.1 ±10.4	35.2 ±12.3	**0.028**	0.345	0.998
**Time of diabetes diagnosis**					1.000
<1 year	2	1	**0.007**	2.485	
1–5 years	0	21		36499.1	
5–10 years	0	15		0.000	
>10 years	3	38		0.000	
**Total Bilirubin, ng/mL,** *mean±SD*	0.24 ±0.10	0.09 ± 0.08	**0.003**	0.000	1.000
**Indirect Bilirubin, ng/mL,** *mean±SD*	0.13 ±0.03	0.02 ± 0.10	**0.010**	1.340	0.998
**Alkaline Phosphatase, IU/L,** *mean±SD*	124 ± 00	63 ±25.56	**0.001**	1.947	0.996

## Discussion

This study showed prevalence rates of HBsAg and anti-HCV in DM2 patients of 0.55% and 2.42%, respectively. HBsAg prevalence was low compared to studies among DM patients in China (13.5%) [19], Gana (5.5%) [25] and Ethiopia (3.7%) [26], but similar prevalence was reported in general population in Brazil [3,6] suggesting that DM2 patients is not at higher risk to infection by HBV than the general population in this region. In addition, no individual had HBV DNA detected in HBsAg positive samples what could be the result of HBV treatment at the time of collection or transportation of samples from distinct regions from Brazil to Southeast region where molecular assays were conducted.

A total of 14.33% of DM2 individuals were immune to HBV (anti-HBs reactive) as it was found in DM2 patients from Southeast region of Brazil (13.7%) [27]. The importance of HBV vaccination in diabetes patients is not much disseminated, although public health policies recommend vaccination through educational campaigns to patients and public health professionals. In 2016, HBV vaccination was extended to all age groups in public health units what could increase the burden of vaccinated individuals in near future.

In group 2, prevalence of anti-HBc and anti-HBs were higher compared to group 1. However, anti-HBs prevalence was still lower than found in Chronic kidney disease (CKD) patients at Northeast region [7] and HIV/HCV infected individuals from Southeast region [28] demonstrating that prevention programs for HBV vaccination should be increase in DM2 patients. Anti-HBc presence was high among older groups showing statistical significance as found in CKD patients, beauticians and general population in Brazil [3,6,7]. The finding of age as a significant variable is probably observed due to the implementation of compulsory vaccination in younger groups in Brazil.

Considering total population, high anti-HCV prevalence (2.42%) was found as the same as demonstrated in DM2 patients from Central West region of Brazil (2 to 2.6%) [21,22], but lower than found in DM2 patients from South region of Brazil (12.9%) [23]. High anti-HCV prevalence was found in group 1 (7.14%) compared to group 2 (1.54%) what could be the reflect of different settings of recruitment or the high frequency of venous access reported by group1 what could be the result of insulin administration in emergency situations, since more than 56% of individuals that reported venous access had more than 10 years of diagnosis of diabetes. We did not know if this venous access was made under universal precaution what could explain the high HCV prevalence in this group. Studies conducted in DM2 patients from Taiwan (6.8%) [29] and Egypt (18%) [17] also observed high anti-HCV prevalence probably due to parenteral exposures from reused needles for vaccinations in these countries.

Anti-HCV reactivity was associated to ALT values in group 2. Korkmaz et al.[30] showed elevated prevalence of HBV and HCV in DM2 patients with increased compared to normal aminotransferase levels (23.1% versus 2.3% for HBV and 19.2% versus 2.0%). In the same group, anti-HCV was not associated to local of residence, although there were not HCV infected individuals from Minas Gerais State (Southeast region).

Anti-HBc and anti-HCV positivity were not associated to risk factors, like history of blood transfusion, sharing razors, hemodialysis and sexual habits in contrast to other studies [15,16,30,31]. On the other hand, anti-HCV seropositivity was associated to high mean age and high mean values of alkaline phosphatase, indirect bilirubin and total bilirubin. Ocak et al.[16] also demonstrated that age was related to HCV seropositivity among DM2 patients on haemodialysis. In this group, individuals were not previously HCV diagnosed cases, but they could have some liver disfunction, like cirrhosis, since bilirubin and alkaline phosphatase elevations were found in this group and associated to anti-HCV positivity. Unfortunately, no information regarding cirrhosis was obtained in this group.

Diabetes duration over 5 years increased the risk of’ HCV infection in the present study as the same as demonstrated among DM2 patients in Saudi Arabia [12]. A long duration of diabetes may lead to the performance of more medical interventions, like exposure to venous access during emergency situations, and may increase the risk of HCV infection.

In this study, most of DM2 patients also presented HCV viremia what could increase the risk of hepatic steatosis, fibrosis and hepatocarcinoma. In addition, these disorders associated with metabolic syndrome, independently increase the risk of cardiovascular and cerebrovascular disease and mortality [32].

This study presents some limitations, such as, the small number of individuals in group 1 for multivariate analysis, absence of information regarding hospitalization and comorbidities and absence of risk factors information and previous HBV treatment in group 2. The design of the study (cross sectional study) cannot demonstrate cause and effect, but it can be used to prove and/or disprove assumptions; captures a specific point in time; contains multiple variables at the time of the data snapshot and the findings and outcomes can be analyzed to create new theories/studies or in-depth research.

In conclusion, low prevalence of HBV and high prevalence of HCV were observed in DM2 patients.

## References

[1] World Health Organization. Hepatitis B. [updated 2017 July; cited 2017 Dec 28]. Available from: http://www.who.int/mediacentre/factsheets/fs204/en/

[2] World Health Organization. Hepatitis C. [updated 2017 October; cited 2017 Dec 28]. Available from: http://www.who.int/mediacentre/factsheets/fs164/en/

[3] Pereira LM, Martelli CM, Merchán-Hamann E, Montarroyos UR, Braga MC, de Lima ML, et al Population-based multicentric survey of hepatitis B infection and risk factor differences among three regions in Brazil. Am J Trop Med Hyg.2009;81: 240–247. 19635877

[4] Pereira LM, Martelli CM, Moreira RC, Merchan-Hamman E, Stein AT, Cardoso MR, et al Prevalence and risk factors of Hepatitis C virus infection in Brazil, 2005 through 2009: a cross-sectional study. BMC Infect Dis. 2013; 13:60 10.1186/1471-2334-13-60 23374914 PMC3574834

[5] Santos Cruz M, Andrade T, Bastos FI, Leal E, Bertoni N, Villar LM, et al Key drug use, health and socio-economic characteristics of young crack users in two Brazilian cities. Int J Drug Policy. 2013;24:432–438. 10.1016/j.drugpo.2013.03.012 23632130

[6] Villar LM, de Paula VS, de Almeida AJ, do Ó KM, Miguel JC, Lampe E. Knowledge and prevalence of viral hepatitis among beauticians. J Med Virol. 2014; 86:1515–21. 10.1002/jmv.23993 24916521

[7] Ribeiro Barbosa J, Sousa Bezerra C, Carvalho-Costa FA, Pimentel de Azevedo C, Lopes Flores G, Baima Colares JK, et al Cross-Sectional Study to Determine the Prevalence of Hepatitis B and C Virus Infection in High Risk Groups in the Northeast Region of Brazil. Int J Environ Res Public Health. 2017;14: pii: E793 10.3390/ijerph14070793 28714924 PMC5551231

[8] Cortes VF, Taveira A, Cruz HM, Reis AA, Cezar JS, Silva BS, et al Prevalence of Hepatitis B and C virus infection among alcoholic individuals: importance of screening and vaccination. Rev Inst Med Trop Sao Paulo. 2017;59:e47 10.1590/S1678-9946201759047 28793018 PMC5626222

[9] International Diabetes Federation. Diabetes Atlas, Eighth Edition. 2017 Cited 2018/08/20. Available from URL: http://www.idf.org/idf-diabetes-atlas-8th-edition.html.

[10] Nascimento Cde M, Mambrini JV, de Oliveira CM, Giacomin KC, Peixoto SV. Diabetes, hypertension and mobility among Brazilian older adults: findings from the Brazilian National Household Sample Survey (1998, 2003 and 2008). BMC Public Health. 2015; 15:591 10.1186/s12889-015-1956-2 26116434 PMC4483209

[11] Garcia-Compean D, Jaquez-Quintana JO, Gonzalez-Gonzalez JA, Maldonado-Garza H. Liver cirrhosis and diabetes: Risk factors, pathophysiology, clinical implications and management. World Journal of Gastroenterology. 2009; 15:280–288. 10.3748/wjg.15.280 19140227 PMC2653324

[12] Ba-Essa EM, Mobarak EI, Al-Daghri NM. Hepatitis C virus infection among patients with diabetes mellitus in Dammam, Saudi Arabia. BMC Health Serv Res. 2016; 16:313 10.1186/s12913-016-1578-0 27464785 PMC4963943

[13] Thompson ND and Perz Joseph F. Eliminating the Blood: Ongoing Outbreaks of Hepatitis B Virus Infection and the Need for Innovative Glucose Monitoring Technologies. J of Diabetes Sci and Technol. 2009; 3:283–288. 20144359 10.1177/193229680900300208 PMC2771515

[14] Cadranel JF, Di Martino V, Lambrey G, Mourlhon C, Nalet B, Anciaux ML, et al Prevalence of hepatitis C infection and risk factors in hospitalized diabetic patients: results of a cross-sectional study. Eur J Gastroenterol Hepatol. 2008; 20:829–836. 10.1097/MEG.0b013e3282fc73a1 18794595

[15] Sotiropoulos A, Peppas TA, Skliros E, Apostolou O, Kotsini V, Pappas SI. Low prevalence of hepatitis C virus infection in Greek diabetic patients. Diabet Med. 1999; 16:250–252. 10227572 10.1046/j.1464-5491.1999.00009.x

[16] Ocak S, Duran N, Kaya H, Emir I. Seroprevalence of hepatitis C in patients with type 2 diabetes mellitus and non-diabetic on haemodialysis. Int J Clin Pract. 2006; 60:670–674. 10.1111/j.1368-5031.2006.00738.x 16805751

[17] Chehadeh W, Kurien SS, Abdella N, Ben-Nakhi A, Al-Arouj M, Almuaili T, et al Hepatitis C virus infection in a population with high incidence of type 2 diabetes: impact on diabetes complications. J Infect Public Health. 2011; 4:200–206. 10.1016/j.jiph.2011.05.004 22000848

[18] Naing C, Mak JW, Ahmed SI, Maung M. Relationship between hepatitis C virus infection and type 2 diabetes mellitus: meta-analysis. World J Gastroenterol. 2012; 18:1642–1651. 10.3748/wjg.v18.i14.1642 22529694 PMC3325531

[19] Lu J, Hou X, Tu H, Tang Z, Xiang Y, Bao Y, et al Hepatitis B Virus Infection Status is more prevalent in patients with type 2 diabetes. J Diabetes Investig. 2017; 8 (4): 619–625. 10.1111/jdi.12609 27930871 PMC5497041

[20] Cai C, Zeng J, Wu H, Shi R, Wei M, Gao Y, et al Association between hepatitis B virus infection and diabetes mellitus: A meta-analysis. Exp Ther Med. 2015; 10:693–698. 10.3892/etm.2015.2537 26622377 PMC4509106

[21] Parolin MB, Réa R, Vargas RM, de Almeida AC, Baldanzi GR, Lopes RW. Prevalence of hepatitis C infection in patients with type 2 diabetes mellitus. Arq Gastroenterol. 2006; 43:77–80. 17119658 10.1590/s0004-28032006000200003

[22] Costa LM, Mussi AD, Brianeze MR, Souto FJ. Hepatitis C as a risk factor for diabetes type 2: lack of evidence in a hospital in central-west Brazil. Braz J Infect Dis. 2008; 12:24–26. 18553010 10.1590/s1413-86702008000100007

[23] Greca LF, Pinto LC, Rados DR, Canani LH, Gross JL. Clinical features of patients with type 2 diabetes mellitus and hepatitis C infection. Braz J Med Biol Res. 2012; 45:284–90. 10.1590/S0100-879X2012007500013 22286533 PMC3854195

[24] Hirakauva EY, Ferraz ML, Perez RM, Ferreira AS, Silva AE, Hauache O, Pestana JO. Prevalence of diabetes mellitus in renal transplant patients with hepatitis B or C virus infection. Transplant Proc. 2002; 34:3220–3222. 12493426 10.1016/s0041-1345(02)03657-6

[25] Ephraim R, Nsiah P, Osakunor D, Adoba P, Sakyi S, Anto E. Seroprevalence of Hepatitis B and C Viral Infections among Type 2 Diabetics: A Cross-sectional Study in the Cape Coast Metropolis. Ann Med Health Sci Res. 2014; 4:719–722. 10.4103/2141-9248.141529 25328781 PMC4199162

[26] Mekonnen D, Gebre-Selassie S, Fantaw S, Hunegnaw A, Mihret A. Prevalence of hepatitis B virus in patients with diabetes mellitus: a comparative cross sectional study at Woldiya General Hospital, Ethiopia. Pan Afr Med J. 2014; 17:40 10.11604/pamj.2014.17.40.2465 24932351 PMC4048674

[27] Arrelias CC, Bellissimo-Rodrigues F, Lima LC, Silva AS, Lima NK, Zanetti ML. Hepatitis B vaccination coverage in patients with diabetes mellitus. Rev Esc Enferm USP. 2016; 50:255–262. 10.1590/S0080-623420160000200011 27384205

[28] Flores GL, de Almeida AJ, Miguel JC, Cruz HM, Portilho MM, Scalioni Lde P, et al A Cross Section Study to Determine the Prevalence of Antibodies against HIV Infection among Hepatitis B and C Infected Individuals. Int J Environ Res Public Health. 2016;13: E314 10.3390/ijerph13030314 26978383 PMC4808977

[29] Chen HF, Li CY, Chen P, See TT, Lee HY. Seroprevalence of hepatitis B and C in type 2 diabetic patients. J Chin Med Assoc. 2006; 69:146–152. 10.1016/S1726-4901(09)70195-9 16689194

[30] Korkmaz H, Kesli R, Onder Pamuk B, Ipekci SH, Terzi Y, Kebapcilar L. Assessment of evidence for positive association and seroprevalence of hepatitis B and C in diabetic patients in a developing country. J Investig Med. 2015; 63:251–257. 10.1097/JIM.0000000000000126 25415060

[31] Gulcan A, Gulcan E, Toker A, Bulut I, Akcan Y. Evaluation of risk factors and seroprevalence of hepatitis B and C in diabetic patients in Kutahya, Turkey. J Investig Med. 2008; 56:858–863. 10.2310/JIM.0b013e3181788d28 18667903

[32] Shiffman ML, Gunn NT. Impact of hepatitis C virus therapy on metabolism and public health. Liver Int. 2017; 37:13–18. 10.1111/liv.13282 28052632

